# Alzheimer’s Disease in Social Media: Content Analysis of YouTube Videos

**DOI:** 10.2196/ijmr.8612

**Published:** 2017-10-19

**Authors:** Weizhou Tang, Kate Olscamp, Seul Ki Choi, Daniela B Friedman

**Affiliations:** ^1^ College of Social Work University of South Carolina Columbia, SC United States; ^2^ Arnold School of Public Health Department of Exercise Science University of South Carolina Columbia, SC United States; ^3^ Arnold School of Public Health Department of Health Promotion, Education, and Behavior University of South Carolina Columbia, SC United States

**Keywords:** Alzheimer’s disease, YouTube, videos, content analysis

## Abstract

**Background:**

Approximately 5.5 million Americans are living with Alzheimer’s disease (AD) in 2017. YouTube is a popular platform for disseminating health information; however, little is known about messages specifically regarding AD that are being communicated through YouTube.

**Objective:**

This study aims to examine video characteristics, content, speaker characteristics, and mobilizing information (cues to action) of YouTube videos focused on AD.

**Methods:**

Videos uploaded to YouTube from 2013 to 2015 were searched with the term “Alzheimer’s disease” on April 30th, 2016. Two coders viewed the videos and coded video characteristics (the date when a video was posted, Uniform Resource Locator, video length, audience engagement, format, author), content, speaker characteristics (sex, race, age), and mobilizing information. Descriptive statistics were used to examine video characteristics, content, audience engagement (number of views), speaker appearances in the video, and mobilizing information. Associations between variables were examined using Chi-square and Fisher’s exact tests.

**Results:**

Among the 271 videos retrieved, 25.5% (69/271) were posted by nonprofit organizations or universities. Informal presentations comprised 25.8% (70/271) of all videos. Although AD symptoms (83/271, 30.6%), causes of AD (80/271, 29.5%), and treatment (76/271, 28.0%) were commonly addressed, quality of life of people with AD (34/271, 12.5%) had more views than those more commonly-covered content areas. Most videos featured white speakers (168/187, 89.8%) who were adults aged 20 years to their early 60s (164/187, 87.7%). Only 36.9% (100/271) of videos included mobilizing information. Videos about AD symptoms were significantly less likely to include mobilizing information compared to videos without AD symptoms (23/83, 27.7% vs 77/188, 41.0% respectively; *P*=.03).

**Conclusions:**

This study contributes new knowledge regarding AD messages delivered through YouTube. Findings of the current study highlight a potential gap between available information and viewers’ interests. YouTube videos on AD could be beneficial if the messages delivered meet users’ needs and provide mobilizing information for further resources. Study findings will be useful to government agencies, researchers, nonprofit organizations that promote information about AD, and those responsible for social media to provide useful and accurate health information for the public.

## Introduction

In 2017, approximately 5.5 million Americans are living with Alzheimer’s disease (AD) and nearly 16 million family members and friends provide over 18 billion hours of unpaid care to those with AD and related dementias [1]. AD is a degenerative brain disease and the most common form of dementia [1,2]. Symptoms of AD can include: difficulty remembering, speaking, swallowing, or walking; apathy; depression; impaired communication; disorientation; confusion; poor judgment; and behavior changes [1]. The common risk factors for AD are older age, having a family history of the disease, and carrying the APOE-ɛ4 gene [3-5]. Projections suggest that as baby boomers continue to age, by 2050 the incidence of AD, economic costs, and caregiver demands related to AD will increase exponentially [1].

Research on messages related to aging, cognitive health, and AD initially focused on traditional media, such as magazines, television news, and newspapers [6-8]. Although media coverage of cognitive health appears to have increased over time, the rise is not commensurate with growing scientific evidence that certain health behaviors can help maintain cognitive health [6]. For example, there is strong evidence that regular physical activity, management of cardiovascular risk factors, a healthy diet, and lifelong learning/cognitive training may reduce the risk of cognitive decline [1,9-10]; however, coverage of physical activity and cardiovascular risk factors in popular media is limited [6]. The recent rise of social media usage creates a new platform for the dissemination of this type of health information to large audiences. Emerging research has examined the impact of social media (specifically Facebook and Twitter) on health issues such as AD, diabetes, hypertension, and cancer [11-17]. YouTube, one of the most popular social media platforms on the Web with over one billion users, allows both individuals and organizations to distribute, search for, watch, share, and comment on user-generated video content [18]. Previous studies have examined messages communicated through YouTube on topics including diabetes, myocardial infarction, Ebola, prostate cancer, anorexia, and organ donation [19-24]; however, less is known about the messages related to AD delivered on the platform. While analyses of AD content in traditional media and on more static websites have been conducted [6-8], this study is the first to examine AD information specifically on YouTube. The following three research questions (RQs) were proposed to explore the characteristics and content of YouTube videos related to AD:

RQ1: What are the characteristics (ie, format, source) of YouTube videos about AD?

RQ2: What content about AD is presented in YouTube videos?

RQ3: What types of YouTube videos about AD are viewed most often by audiences?

In 2013, the Alzheimer’s Association and Centers for Disease Control and Prevention (CDC) published *The Healthy Brain Initiative: The Public Health Road Map for State and National Partners* (hereafter referred to as the Public Health Road Map) [25]. This Road Map provides guidance for state and local public health agencies and partners in several public health domains: monitor and evaluate, educate and empower, develop policy and mobilize partnerships, and assure a competent workforce [25]. This document also serves as a directive for researchers as they strive to connect their work to the public sphere. RQs 4 and 5 below are guided by two Road Map action items of the *educate and empower* domain: identify culturally appropriate strategies (E01) and provide links to accurate websites about AD (E03). AD prevalence varies by race. Older African Americans and Hispanics have a higher incidence of AD compared with older whites [1]; however, less is known about whether YouTube provides culturally appropriate AD messages for different ethnic groups.

RQ4: What are the characteristics of speakers appearing in YouTube videos about AD?

RQ5: Do YouTube videos about AD include mobilizing information (ie, website Uniform Resource Locators [URLs], physical addresses, and phone numbers)?

## Methods

### Sample

This content analysis focused on videos posted on YouTube after the release of the Public Health Road Map [25]. We searched YouTube using the term “Alzheimer’s Disease” on April 30^th^, 2016. We included all videos uploaded between January 1^st^, 2013 and December 31^st^, 2015. If a video with the same title or a slightly different title with identical content appeared multiple times, the video was counted as one case and only the earliest video was included. Once duplicates were removed, a total of 478 videos remained. Videos were excluded if they were not in English, lacked an audio component, were longer than 10 minutes, were portions of major motion pictures, or had technical problems (eg, interrupted audio, unclear sound). A total of 271 videos were included for coding and analysis.

### Variable Coding

The codebook for this analysis was adapted from a previous analysis of YouTube videos on organ donation [26]. Variables included in the codebook were also guided by the RQs and action items from the Public Health Road Map [25].

#### Video Characteristics

To answer RQs 1 and 3, the date when a video was posted, URL, video length, and number of views, likes, and dislikes were coded first at the time of capture. After viewing the videos, variables were coded as having a specific characteristic if it was mentioned or appeared at least once. The format of a video may influence whether people view it, like it, and how messages are delivered through it [27]. We categorized video formats into *public service announcement* (PSA), *testimonial*, *news story and interview*, *nonnews interview*, *formal presentation*, *informal presentation*, and *others*. Videos recording a caregiver’s experience or care recipient’s life were coded as a *testimonial*. *News story and interview* refers to videos that are journalistic-style or broadcast on a news program. Interviews conducted in a studio like “Good Morning America” were also coded as *news story and interview.Nonnews interviews* were videos made professionally, but not journalistic in nature, regardless of the appearance of interviewers. Videos in which researchers presented their research findings or informed knowledge about AD formally with slides or at an event (eg, conference, forum, webinar) were coded as a *formal presentation*. Other presentations or informative speeches made by laypersons, students, or those with a computerized voice were coded as an *informal presentation*.

Authorship was defined by “Who posted the video,” which was determined by the user name under the video, and was categorized into individual researcher/health professional, government agency, layperson, nonprofit organization or university, for-profit company or organization, news source (eg, CNN or Fox News), and others that could not be categorized. The type of author was examined by the authors’ profile pages on YouTube. If needed, we searched online for the organization/company name.

#### Video Content

To answer RQ2, video content was categorized into AD awareness, causes of AD, AD symptoms, protective and risk factors, treatment, screening/diagnosis, quality of life of people with AD, caregiving experience, resources of support, clinical trial research, and others.

#### Speaker Characteristics

To answer RQ4, characteristics of speakers that appeared in the videos were coded. A speaker was defined as a person who was shown onscreen and who spoke at least once. Characteristics of each speaker included sex (male, female), race (white, African American, Asian/Pacific Islander, Hispanic, other), age group (adult, aged 20 years to early 60s; older adult, aged 65 years or older), and role. The role of each speaker was identified by his/her title and self-introduction, including people with AD, informal caregivers (eg, family members, friends), journalists or interviewers (eg, reporters, talk show hosts), organization representatives (eg, volunteers, staff), healthcare professionals (eg, physicians, nurses, specialists), actors, researchers, and others (including those unknown/unclear from the video). When a person’s voice was heard in the video, but he/she was not seen, the person was considered a voiceover instead of a speaker.

#### Mobilizing Information

*Mobilizing information* is defined as a particular type of information that permits citizen action [28]. Mobilizing information includes: names, addresses, and phone numbers of sources; titles of documents; specific dates; places of meetings; and website links that can direct the audience to additional resources. To answer RQ5, we examined whether a video included website URLs, physical addresses, or phone numbers for more information.

### Interrater Reliability

Two authors (WT, KO) independently viewed and coded the same 20% random sample of the videos (56/271) to determine interrater reliability. The Cohen’s kappa statistic, which measures interrater agreement of categorical variables between two coders [29], was calculated for all variables. Interrater reliability for all variables ranged from .736 to .964, indicating *almost perfect* coder agreement [30]. One of the coders (WT) finished coding the remainder of the videos.

### Analysis

We analyzed data using SPSS 19.0 (IBM Statistics for Windows, Armonk, New York). Nonparametric frequencies and percentages were calculated for all variables. Associations between video characteristics and content, and between video characteristics and mobilizing information, were examined with Chi-square tests or Fisher’s exact tests. The level of significance was set to *P*<.05.

## Results

Of the 271 videos included in the study, 249 videos were uploaded in 2015, 17 were uploaded in 2014, and 5 were uploaded in 2013. The number of views at the time of data collection ranged from 2 to 431,079, with a mean of 9,876.9 and a median of 166.0. Both mean and median length of videos were approximately 3 minutes.

### RQ1: Video Characteristics

#### Video Format

Informal presentations comprised 25.8% (70/271) of all videos, followed by nonnews interviews (52/271, 19.2%), news stories and interviews (50/271, 18.5%), formal presentations (26/271, 9.6%), testimonials (20/271, 7.4%), and PSAs (15/271, 5.5%). Other types of videos were mostly cartoon episodes (Table 1).

**Table 1:** Video characteristics, content, and speaker’s characteristics of videos related to Alzheimer’s disease on YouTube.

Variables			n (%)	
Video Characteristics^a^				
	**Video Format**			
		Informal presentation		70 (25.8)
		Nonnews interview		52 (19.2)
		News story and interview		50 (18.5)
		Formal presentation		26 (9.6)
		Testimonial		20 (7.4)
		Public service announcement		15 (5.5)
		Other		38 (14.0)
	**Video Authorship**			
		Nonprofit organization/university		69 (25.5)
		Layperson		57 (21.0)
		For-profit organization/company		51 (18.8)
		News source		39 (14.4)
		Researcher		9 (3.3)
		Government agency		5 (1.8)
		Others		41 (15.1)
	**Video Content^a,b^**			
		AD symptoms		83 (30.6)
		Causes of AD		80 (29.5)
		Treatment		76 (28.0)
		Protective and risk factors		50 (18.5)
		AD awareness		45 (16.6)
		Resources of support		41 (15.1)
		Caregiving experience		35 (12.9)
		Quality of life of people with AD		34 (12.5)
		Screening/diagnosis		33 (12.2)
		Clinical trials		32 (11.8)
		Others		41 (15.1)
**Speaker Characteristics^c^**				
	**Sex**			
		Female		124 (66.3)
		Male		126 (67.4)
	**Age Group**			
		Adult (aged 20 years to early 60s)		164 (87.7)
		Older adult (aged 65 years or older)		55 (29.4)
	**Race**			
		White		168 (89.8)
		African American		11 (5.9)
		Asian/Pacific Islander		10 (5.3)
		Other		12 (6.4)
	**Speaker Role**			
		Researcher		72 (38.5)
		Informal caregiver		43 (23.0)
		Journalist/interviewer		43 (23.0)
		Healthcare professional		42 (22.5)
		Organization representative		39 (20.9)
		Individual with AD		23 (12.3)
		Actor		7 (3.7)
		Other		24 (12.8)

^a^Percentage was calculated for 271 videos

^b^Sum may not be 100%, because more than one content area was often covered in a video

^c^One video may include more than one speaker; each speaker was coded separately for his/her sex, race, age group, and role; percentage was calculated for 187 videos with speakers

#### Video Authorship

Approximately 25% of videos (69/271) were posted by nonprofit organizations or universities such as Emory University (11/69), the Alzheimer’s Association (National: 5/69; Chapters: 3/69), and Alzheimer’s Disease International (6/69). Laypersons posted 21.0% of videos (57/271). Fewer videos were posted by for-profit organizations or companies (51/271, 18.8%,), news sources (39/271, 14.4%), individual researchers (9/271, 3.3%), government agencies (5/271, 1.8%), and others (41/271, 15.1%; Table 1). Video format differed according to video authorship. News sources mainly posted news stories and interviews (33/39, 84.6%); 60.0% (3/5) of videos posted by government agencies were formal presentations; and 46.4% (32/69) of videos that nonprofit organizations or universities posted were nonnews interviews (*P*<.001, Fisher’s exact test).

### RQ2: Video Content

The most common focus of the videos was AD symptoms (83/271, 30.6%). Changes in behavior, thinking, personality, and mood were presented as common symptoms of AD. Almost one third of videos (80/271, 29.5%) presented causes of AD such as beta amyloid accumulation, plaque formation, and tangles. AD treatment, including therapies and programs aimed at helping people with AD stay physically, mentally, and socially active, were introduced in 28.0% of videos (76/271). Approximately 18.5% of videos (50/271) presented protective (eg, regular physical activity, healthy diets) or risk (eg, high level of stress, low level of vitamin D) factors (Table 1). Approximately 26.6% (72/271) of the videos presented caregiving-related information. Among those videos about caregiving, 66.7% (48/72) included challenges that caregivers may face (future plan, financial burden, searching for missing care recipients) and 43.1% (31/72) included available resources for caregivers (organizations that provides information, caregiver support groups and programs). Half of the videos about caregiving provided information on instrumental support, including financial resources and caregiving skills (36/72, 50.0%); 29 videos (29/72, 40.3%) described psychological perspectives (distress facing care recipients’ symptoms, worry about genetic predisposition to AD) and 27 videos (27/72, 37.5%) were about social support (help and support from organizations, friends, family members, and other caregivers; social activities). Only 4 caregiving videos (4/72, 5.6%) presented physical needs of caregivers. Table 2 provides detailed information on video content with specific examples.

We examined the association between video content and format. Significantly higher percentages of formal presentations (13/26, 50.0%) and informal presentations (34/70, 48.6%) included causes of AD, compared with news stories and interviews (13/50, 26.0%), nonnews interviews (13/52, 25.0%), and other formats (7/38, 18.4%; χ^2^=35.2, degrees of freedom [df]=6; *P*<.001). Similarly, AD symptoms were addressed significantly more often in informal presentations (31/70, 44.3%) and formal presentations (10/26, 38.5%) compared with other video formats (χ^2^=16.1, df=6; *P*=.01). PSAs were significantly more likely to include resources of support (6/15, 40.0%) compared with testimonials (5/20, 25.0%), news stories and interviews (12/50, 24.0%), formal presentations (4/26, 15.4%), nonnews interviews (6/52, 11.5%), and other formats (7/38, 18.4%; *P*<.001, Fisher’s exact test).

**Table 2:** Content of videos related AD on YouTube.

Content		Examples
AD awareness		Facts about AD (prevalence, cost of caring annually), support for AD-related research, fundraising, dignity in mental health, stigma about AD
Cause of AD		Proteins in brain, beta amyloid accumulation, tau protein accumulation, tangles, plaque formation, genetic risk factors (APOE-ɛ4)
AD symptoms		10 early signs and symptoms of Alzheimer’s by Alzheimer’s Association; changes in thinking, behavior, personality, and mood; decline in the sense of smell
Protective factors		Regular physical activity, heart health, healthy diets
Risk factors		High level of stress, low level of vitamin D
Screening/diagnosis		Importance of early identification, memory test, cognitive test, magnetic resonance imaging scan of brain, positron emission tomography–computed tomography blood test, biomarkers
Treatment		Programs aimed at helping people with AD communicate with partners; stay active physically, mentally, and socially; new and promising drugs with positive testing results; treatments available for symptoms
Quality of life of people with AD		Personal stories about early symptoms, diagnosis, and experience of AD; simulation of AD experience
Caregiving experience		Daily tasks, emotions, and feelings (scared, sad, cry) of caregivers
Resources of support		Introduction of support programs and research centers, resources for caregivers, social activity, and campaigns such as “Walk to End Alzheimer’s”
Clinical trials		Research studies with mice models investigating causes of AD, risk factors, treatment, and comparison of the brain of AD to the one without AD
Other		Graduate students’ introduction about their research topics and why they entered the field, faith related (bible code, deliverance), history of discovering AD, evaluating accuracy of information in a movie
**Caregiving-related information**		
	Challenges	Future plan, financial burden, searching for missing care recipients
	Available resources	Organizations that provide information, caregiver support groups and programs
	Support	Instrumental support (financial resources, caregiving skills); psychological support (distress facing care recipients’ symptoms, worry about genetic predisposition to AD); social support (help and support from organizations, friends, family members, and other caregivers; social activities)

We also examined the association between video content and authorship. Videos from news sources (9/39, 23.1%) and laypersons (12/57, 21.1%) were significantly more likely to discuss the quality of life of people with AD than for-profit companies (7/51, 13.7%), nonprofit organizations or universities (5/69, 7.2%), or other sources (1/41, 2.4%; *P*=.02, Fisher’s exact test). Other video content was not significantly associated with authorship (data not shown).

### RQ3: Audience Engagement

Videos posted earlier on YouTube had more views than those posted later; however, the majority of videos analyzed were posted in 2015 (249/271, 91.9%). There were no significant differences regarding video characteristics or content across the three years examined. YouTube users viewed testimonials most often compared to other format types, with a mean number of 41,916 views. Quality of life of people with AD, caregiving experience, causes of AD, treatment, and AD symptoms were the main content areas that ranked highly in mean number of views. In terms of authorship, videos posted by news sources (mean number of views per video=11,774.7) were viewed the most, followed by nonprofit organizations or universities (mean=10,100.5) and laypersons (mean=7239.0; Table 3). Most YouTube viewers of the videos analyzed did not click on *like* or *dislike*. For most of the videos, the number of likes was less than 5% of the total number of views (266/271, 98.2%; data not shown). Over half of the videos did not have any dislikes (201/271, 74.2%). In an ad hoc analysis, the number of comments for each video was coded in August 2017. There were 3020 comments left by users for a total of 239 videos (range=0-716, mean=12.6; comments were disabled for 12 videos; 20 videos were not available for viewing at the time of coding). Videos posted by others (authors could not be categorized) received the highest number of comments (mean=47.6), followed by laypersons (mean=17.6), researchers/health professionals (mean=6.4), nonprofit organizations (mean=6.4), and news sources (mean=4.7). Videos with content regarding quality of life of individuals with AD received the highest number of comments (mean=31.0).

**Table 3:** Audience engagement by video characteristics.

Video Characteristics			Views			Range		
			Mean	Total		Minimum		Maximum
**Video format**								
	Testimonial (n=20)	41,916.2		838,324	31		431,079	
	Public service announcement (n=15)	28,306.5		424,597	9		170,984	
	Formal presentation (n=26)	8286.6		215,451	12		118,245	
	Informal presentation (n=70)	4840.2		338,815	12		271,076	
	Nonnews interview (n=52)	3468.1		180,343	8		37,920	
	News story and interview (n=50)	989.3		49,465	8		17,514	
	Other (n=38)	16,569.4		629,637	2		399,971	
**Video content**								
	Quality of life of people with AD (n=34)	29,739.2		1,011,134	8		431,079	
	Caregiving (n=35)	18,121.3		634,245	8		431,079	
	Causes of AD (n=80)	13,845.8		1,107,662	12		399,971	
	Treatment (n=76)	8715.4		662,372	12		399,971	
	AD symptoms (n=83)	8321.6		690,691	8		399,971	
	Resources of support (n=41)	7606.6		311,870	2		123,340	
	AD awareness (n=45)	6639.1		298,760	8		123,340	
	Protective and risk factor (n=50)	1782.1		89,106	13		15,155	
	Screening/diagnosis (n=33)	985.6		32,526	12		13,696	
	Clinical trials (n=32)	857.2		27,431	17		17,514	
	Others (n=41)	12,073.1		494,998	2		271,076	
**Authorship**								
	News source (n=39)	11,774.7		459,213	8		431,079	
	Nonprofit organization/university (n=69)	10,100.5		696,933	8		170,984	
	Layperson (n=57)	7239.0		412,622	12		382,509	
	Profit organization (n=51)	3200.9		163,245	15		38,962	
	Researcher/health professional (n=9)	1886.1		16,975	12		15,155	
	Government agency (n=5)	1065.0		5325	21		1912	
	Others (n=41)	22,495.6		922,319	2		399,971	

### RQ4: Speaker Characteristics

There were 84 videos (84/271, 31.0%) without a speaker, which only had voiceovers. Among the videos with at least one speaker (187/271), male and female speakers were equally likely to appear in the videos (126/187, 67.4% male; 124/187, 66.3% female, respectively). Most videos featured white speakers (168/187, 89.8%) who were adults aged 20 years to their early 60s (164/187, 87.7%). Researchers appeared in the videos most often (72/187, 38.5%), followed by informal caregivers (43/187, 23.0%), journalists or interviewers (43/187, 23.0%), healthcare professionals (42/187, 22.5%), organization representatives (39/187, 20.9%), people with AD (23/187, 12.3%), and actors (7/187, 3.7%). Other speaker roles (24/187, 12.8%) included students, celebrities, congressmen, and those who did not introduce themselves (Table 1).

### RQ5: Mobilizing Information

All videos failed to include a physical address (271/271, 100.0%) and 91.9% (249/271) of videos did not include phone number for viewers to locate additional information. Approximately 36.9% (100/271) of videos included a website URL. These links were often to *.com* (45/100) and *.org* (38/100) websites. Only 4 videos had a *.gov* URL. Video authorship was significantly associated with the presence of mobilizing information. More videos posted by nonprofit organization or university (35/69, 50.7%), for-profit organization or company (25/51, 49.0%), researcher/health professional (4/9, 44.4%), and government agencies (2/5, 40.0%) included a website URL compared with those posted by news sources (9/39, 23.1%), laypersons (11/57, 19.3%), and others (14/41, 34.1%; *P*=.002, Fisher’s exact test; Table 4).

**Table 4:** Mobilizing information by authorship and video content (AD symptoms, resources of support).

			Videos with mobilizing information, n=100	Videos without mobilizing information, n=171	χ^2^	Degrees of freedom	P-value
			n (%)	n (%)			
**Authorship^a^**					-	-	.002
	Nonprofit organization/university		35 (50.7)	34 (49.3)			
	For-profit organization/company		25 (49.0)	26 (51.0)			
	Layperson		11 (19.3)	46 (80.7)			
	News source		9 (23.1)	30 (76.9)			
	Researcher/health professional		4 (44.4)	5 (55.6)			
	Government agency		2 (40.0)	3 (60.0)			
	Others		14 (34.2)	27 (65.8)			
**Video content^b^**							
	**AD Symptoms**				4.3	1	.03
		Yes	23 (27.7)	60 (72.3)			
		No	77 (41.0)	111 (59.0)			
	**Resources of Support**				23.7	1	<.001
		Yes	29 (70.7)	12 (29.3)			
		No	71 (30.9)	159 (69.1)			

^a^Fisher’s exact test

^b^Associations between presence of mobilizing information and all other video content topics not presented here were not significant

We examined the relationship between mobilizing information and video content and found that videos focusing on providing resources of support were significantly more likely to include a website URL than videos that did not focus on resources of support (29/41, 70.7% vs 71/230, 30.9% respectively; χ^2^=23.7, df=1; *P*<.001). Videos about AD symptoms were significantly less likely to include mobilizing information compared to videos without AD symptoms (23/83, 27.7% vs 77/188, 41.0% respectively; χ^2^=4.3, df=1; *P*=.03). Videos focusing on other content categories were not associated with the presence of mobilizing information.

## Discussion

Guided by action items from the Public Health Road Map [25], the current study examined the characteristics and content of messages about AD delivered through social media based on 271 videos uploaded to YouTube between 2013 and 2015. Findings of this study demonstrated that most videos included multiple content areas, with AD symptoms, causes of AD, and treatment being commonly addressed. However, videos with these frequently covered contents had fewer mean numbers of views than those videos focusing on quality of life and caregiving. Speakers featured in these videos were mostly white adults from the ages of 20 years to their early 60s. Less than half of the videos included mobilizing information.

The majority of videos analyzed were posted in 2015. Several reasons are possible for this result. First, much attention was paid to AD and dementia between late 2014 and 2015. For example, the 2015 government spending package, known as the *cromnibus*, included an increase of US $25 million for the National Institute on Aging, with an expectation that much of the funding would support additional research on AD and dementia. This funding may have influenced media coverage about AD and encouraged others to post videos online about AD. Another reason for more video postings in 2015 may be that older videos (published prior to 2015) may have become unavailable due to a terminated account associated with that video, copyright infringement, and uploader’s removal. Since the data was collected in early 2016, those videos posted in 2015 are more likely to remain available than videos posted in 2014 and earlier.

Results demonstrated that most videos addressed multiple content areas. The primary foci of the videos were AD symptoms, causes of AD, treatment, and protective and risk factors. Fewer videos were focused on diagnosis and early screening. This finding is consistent with previous studies on cognitive health messages in television news, magazines, and newspapers [6-8]. This result also indicates that the information on early diagnosis and screening did not receive much attention by the individuals or organizations who created the videos, even though this information would be important for the general public [31-33].

The videos with frequently covered content such as AD treatment, symptoms, and protective and risk factors had fewer mean numbers of views than those focused on quality of life and caregiving. Videos regarding quality of life of individuals with AD also received the most comments. The number of views and comments metric can reflect viewers’ interests. Therefore, fewer views of videos containing the most commonly featured content highlight a potential gap between available information and viewers’ interests. Although we do not have the profile of viewers, people seeking AD information on YouTube are possibly caregivers of people with AD, based on the focus of videos that ranked high in number of views. Our results also showed that testimonial videos have the highest views among all formats. This finding indicates that YouTube users preferred to view testimonial videos that presented experiences of AD patients and their families. Previous research indicates that people prefer receiving health information through testimonials, thus the use of testimonials to distribute health information can be beneficial for health decision-making and behavior change [34,35]. However, AD videos using testimonials were less available on YouTube in this study. Future videos need to consider providing AD information in a testimonial format to increase viewership of this content. Understanding the viewers and their needs will be important for nonprofit organizations, researchers, and government agencies who plan to provide information and resources on YouTube. Creating content that people need will be beneficial to both the viewers and the uploaders in terms of marketing and health promotion strategies. Further research is needed to understand people using YouTube for AD information and messages that can reach different target audiences (caregivers, people with AD, and the general public), and to provide appropriate information that meets viewers’ needs.

Speakers shown in YouTube videos related to AD were mostly white adults, despite the high usage of video-sharing sites among African Americans and Hispanics [36]. Considering the high incidence of AD among African Americans and Hispanics [37-41], the current AD messages on YouTube may not address specific information for, and cultural aspects of, these high-risk populations. Health messages tailored to match individual characteristics or targeted to specific group characteristics are beneficial to enhance people’s attention and involvement in health issues [42,43]. In addition, most speakers portrayed in videos were adults from the ages of 20 years to their early 60s, and few speakers were individuals with AD or informal caregivers. Most people living with AD are aged 75 years or older, and approximately one in three informal caregivers who need information and support are aged 65 years or older [1]. People who create and upload YouTube videos may not be similar in demographics to those who are viewers of YouTube content, thus the videos may not reflect unique needs of the users, especially aging-related needs. Action items within the Public Health Road Map support the importance of identifying and promoting culturally appropriate strategies to increase public awareness about AD. Future research is needed to develop culturally appropriate messages for diverse populations and to understand the effectiveness of those messages delivered through YouTube and other social media.

Only 36.9% (100/271) of AD videos on YouTube included a website URL and most videos did not include a physical address or phone number. Providing mobilizing information is an important strategy that helps connect populations to additional resources [44]. AD videos focused on providing resources of support were more likely to include a website URL, which is an expected finding given the purpose of those videos. Videos focused on AD symptoms were less likely to include a website URL, although viewers may need contact information of organizations and health professionals for further screening and diagnosis when they have similar symptoms to those presented in the video. Providing mobilizing information also helps to promote organizations, including news sources and nonprofit organizations, for directing users to further health information and engaging them in advocacy activities [44,45]. All types of organizations (ie, nonprofit, for-profit, and government agencies) are increasingly turning toward social media to spread organizational news [46]. Using YouTube can be a wise marketing and public relations strategy for organizations who create videos to reinforce awareness of their programs and services, promote their fundraising efforts, and shape their organizational brand and identity [46,47].

Action items of the Public Health Road Map suggest the importance of disseminating evidence-based messages about risk reduction for preserving cognitive health [25]. Although we did not assess accuracy of information that AD-related YouTube videos provide, we noticed in an ad hoc analysis that 22 videos (either title or content) presented seemingly inaccurate or misleading information. The inaccurate or misleading information included the following: AD is contagious, anti-anxiety drugs cause AD, particular foods/drinks can be used to treat AD, and that certain vaccines can slow down the progression of AD. Those videos were mostly posted by laypersons or other authors who could not be categorized. As with most online information, the content on YouTube may not be peer-reviewed; therefore, registered users can post any content. Studies on other health topics presented on YouTube showed that videos often have inaccurate or misleading information [20-24,48]. Future research is needed to evaluate the quality of information provided by videos related to AD using in-depth methods, and to develop measures that can help audiences evaluate the information presented on YouTube and other social media platforms.

### Limitations

This study had some limitations. First, we only used the single search term “Alzheimer’s disease”, thus we might have missed some videos focused on AD using other search terms such as “Alzheimer”. Second, we only analyzed videos in English and videos about AD in other languages would have been missed. Finally, we also excluded videos over 10 minutes in length, based on other similar studies [22,26]. Previous research showed that most YouTube videos are less than 10 minutes long, and people are mainly interested in viewing these types of shorter videos [49,50].

### Conclusions

This is the first study to analyze YouTube videos about AD, and it contributes new knowledge regarding AD messages delivered through this popular platform. Findings of this study also respond to action items in the Public Health Road Map [25] by attempting to understand whether AD messages on YouTube are culturally appropriate and include website URLs for further resources. The Public Health Road Map provides guidance for state and local public health agencies and partners in several public health domains to monitor and evaluate the status of cognitive health, including AD and related programs, and educate and empower the public and relevant agencies [25]. This study identified a lack of mobilizing information, as well as a potential gap between information available and viewers’ interests, which will be useful for government agencies, researchers, and nonprofit organizations that promote information about AD (and those responsible for social media) to provide useful and accurate AD information for the public. Future research is needed to assess whether messages disseminated on YouTube are evidence-based and to understand viewers’ attitudes toward this information based on their comments. YouTube can be a useful platform to deliver AD information to reach high-risk populations; however, videos need to be improved in terms of cultural appropriateness of the information, users’ characteristics and interests, and accuracy. AD videos could be more beneficial if they deliver messages that meet users’ needs and include mobilizing information that can direct people to relevant and credible resources.

## Abbreviations

AD: Alzheimer’s disease
CDC: Centers for Disease Control and Prevention
df: degrees of freedom
PSA: public service announcement
RQ: research question
URL: Uniform Resource Locator
